# Private Health Sector in India-Ready and Willing, Yet Underutilized in the Covid-19 Pandemic: A Cross-Sectional Study

**DOI:** 10.3389/fpubh.2020.571419

**Published:** 2020-10-16

**Authors:** Samira Davalbhakta, Supriya Sharma, Shefali Gupta, Vishwesh Agarwal, Gaurav Pandey, Durga Prasanna Misra, Bijaya Nanda Naik, Ashish Goel, Latika Gupta, Vikas Agarwal

**Affiliations:** ^1^Byramjee Jeejeebhoy Government Medical College and Sassoon General Hospitals, Pune, India; ^2^Department of Surgical Gastroenterology, Sanjay Gandhi Postgraduate Institute of Medical Sciences, Lucknow, India; ^3^Department of Microbiology, Maharishi Markandeshwar Institute of Medical Sciences and Research (MMIMSR), Mulana, India; ^4^Mahatma Gandhi Missions Medical College, Navi Mumbai, India; ^5^Department of Gastroenterology, Sanjay Gandhi Postgraduate Institute of Medical Sciences, Lucknow, India; ^6^Department of Clinical Immunology and Rheumatology, Sanjay Gandhi Postgraduate Institute of Medical Sciences, Lucknow, India; ^7^Department of Community Medicine, NAMO Medical Education and Research Institute, Silvassa, India; ^8^Department of Medicine, University College of Medical Sciences, New Delhi, India

**Keywords:** public-private sector partnerships, delivery of health care, private healthcare sector, pandemics, COVID-19, public health, India

## Abstract

**Background:** The private medical sector is a resource that must be estimated for efficient inclusion into public healthcare during pandemics.

**Methods:** A survey was conducted among private healthcare workers to ascertain their views on the potential resources that can be accessed from the private sector and methods to do the same.

**Results:** There were 213 respondents, 80% of them being doctors. Nearly half (47.4%) felt that the contribution from the private medical sector has been suboptimal. Areas suggested for improved contributions by the private sector related to patient care (71.8%) and provision of equipment (62.4%), with fewer expectations (39.9%) on the research front. Another area of deemed support was maintaining continuity of care for non-COVID patients using virtual consultation services (77.4%), tele-consultation being the preferred option (60%). 58.2% felt that the Government had not involved the private sector adequately; and 45.1% felt they should be part of policy-making.

**Conclusion:** A streamlined pathway to facilitate the private sector to join hands with the public sector for a national cause is the need of the hour. Through our study, we have identified gaps in the current contribution by the private sector and identified areas in which they could contribute, by their own admission.

## Introduction

**Figure 1:**
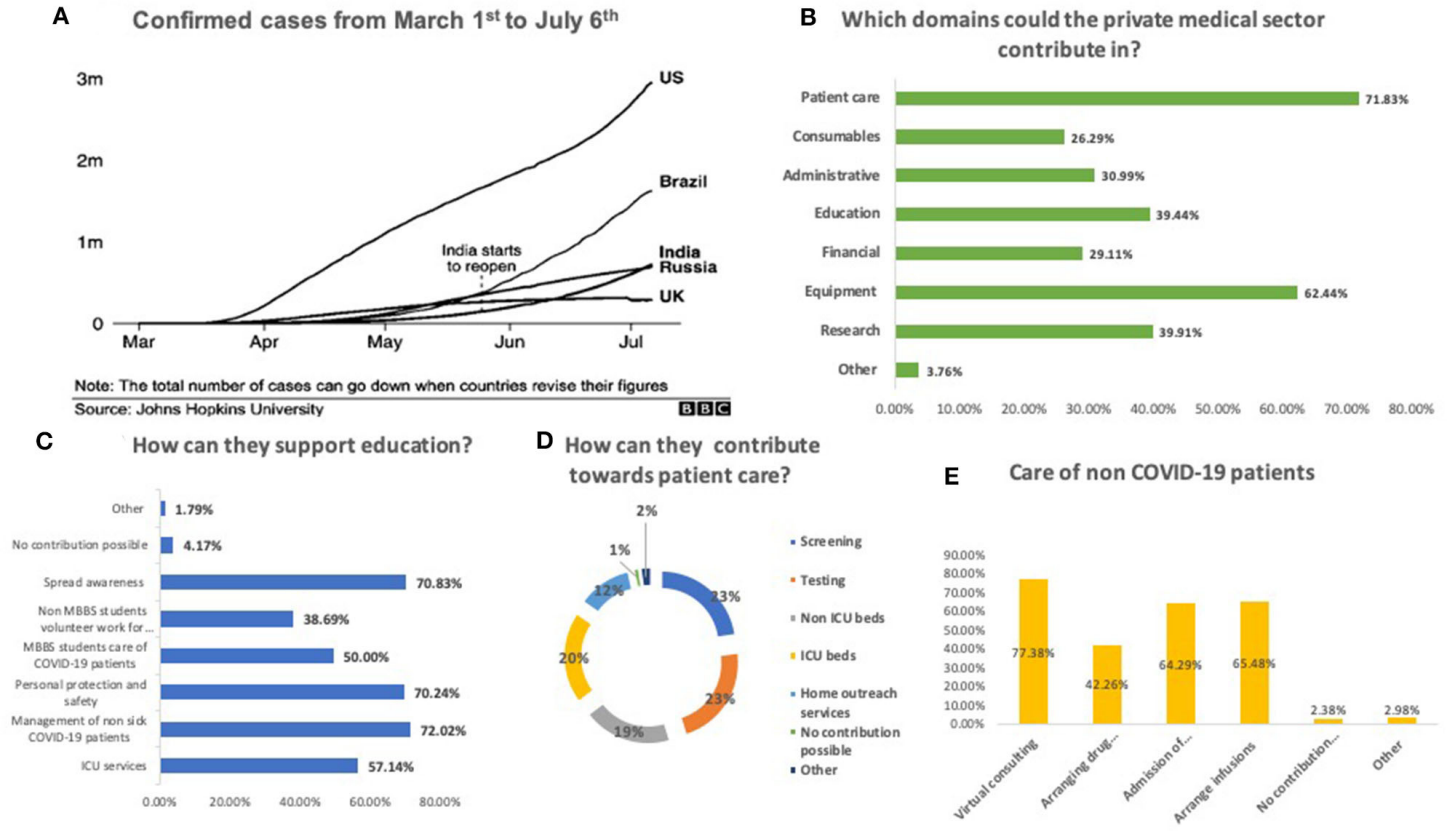
Extent of the COVID-19 pandemic and willingness of the private sector to contribute toward the pandemic. **(A)** Confirmed cases from 1st March'20 to 6th July ‘20. **(B)** Domains that the private sector could contribute in. **(C)** How they can support education. **(D)** How they can contribute toward patient care. **(E)** How they can care for no COVID-19 patients.

The novel coronavirus disease (COVID-19) has consumed and exhausted widespread national health resources with unprecedented speed, and is expected to leave lasting consequences on global health, economy and growth (1). The massive losses (2) call for the amalgamation of rapid innovations alongside bold public health measures led by a courageous political will to tackle this unique “*War sans Weapon”* situation (3). As of May 28th 2020, India has reported 158,332 COVID-19 cases (4), a number that is rapidly rising, consuming the public healthcare system, which has been at the fore of this pandemic, despite deficient infrastructure, manpower, and poor resources (5, 6). Amongst other countries, India currently ranks third with regards to the daily increase in the cases (Figure 1A) (7). With an availability of 0.55 public-hospital beds to 1,000 population (8), it is not unreasonable to expect that the public sector may not be able to provide effective, sustained and uninterrupted healthcare in the face of the rising numbers. Not surprisingly, countries ahead of us on the pandemic curve have recognized the need to utilize all available healthcare resources, forging partnerships between public and private healthcare sectors.

In India, the healthcare scenario has transformed over the last few decades (9, 10), and almost 87% services are provided in the private sector, making it a major stakeholder (11). The first decade of this century saw a growth in private sector beds by almost 70%, bringing their total share to nearly 63% (12). Although healthcare professionals in private enterprises are best suited to provide insights into potential areas of access from the private sector and methods to do so, yet there voices are seldom heard in the scientific world. Improvements in outcomes and health indicators have been reported after private-public partnerships (PPP) in previous reports (13). The National Health Policy (NHP) 2017 not only advocates for exploring role of PPP in achieving Universal Health Coverage (UHC) (14), but PPP has also been proposed as an efficient model for disaster risk reduction (15, 16).

Moreover, the success of teleconsultation based services among private sector healthcare workers in Australia and Canada suggest possibly greater support in terms of infrastructure and participation from the private sector regarding this field (17, 18). The present survey was conducted to explore the opinions and preparedness of healthcare workers (HCWs) in the private sector, on public-private partnerships (PPP) and the feasibility and willingness to participate in teleconsultation services to provide sustained and uninterrupted healthcare response in the face of the current pandemic.

## Materials and Methods

### Study Population Selection

An online survey was conducted in April 2020, and a pre-tested, content validated questionnaire was circulated over WhatsApp® groups of healthcare professionals (doctors, nurses, technicians, students and administrators amounting to nearly 2,000 individuals) in the private hospitals across India (19). The private sector was defined as any hospital or individual in practice not funded by the state, and providing in-patient or out-patient services. This includes corporate hospitals (both teaching and non-teaching facilities), non-profit, faith based, non-government organizations, and individual as well as small scale private enterprises (e.g., nursing homes, day care facilities, clinics etc.). The participants were requested to provide an informed consent at the beginning of the survey. We did not offer incentives for participation.

### Survey Design

The questionnaire (Table 1) featured 20 questions, of which five identified respondent characteristics. Fourteen items were multiple choice, with one being open-ended.

**Table 1:** Survey responses.

**Question**	**Response [*n* (%)]**
**Q1. Do you think the private medical sector in India is contributing enough toward the COVID-19 pandemic? [single response**, ***N*** **=** **213]**	
No	101 (47.42%)
Yes	74 (34.74%)
I am not sure	38 (17.84%)
**Q2. Which domains do you think the private medical sector could contribute in?[multiple responses**, ***N*** **=** **213]**	
Research	85 (39.91%)
Patient care	153 (71.83%)
Financial	62 (29.11%)
Education	84 (39.44%)
Administrative (e.g., Formulation and execution of guidelines)	66 (30.99%)
Consumables	56 (26.29%)
Equipment (e.g. ventilators)	133 (62.44%)
Other (please specify)	8 (3.76%)
**Q3. How do you think the private sector could contribute toward research?[multiple responses**. ***N*** **=** **70)**	
Develop new drugs	39 (55.71%)
Repurpose existing drugs	39 (55.71%)
Develop new methods of testing	50 (71.43%)
Develop a vaccine	41 (58.57%)
Not sure	4 (5.71%)
Other (please specify)	4 (5.71%)
**Q4. How do you think the private sector could contribute toward patient care?[Multiple responses**, ***N*** **=** **168]**	
Screening services	117 (69.64%)
Testing	116 (69.05%)
Offer non-ICU beds for admission	100 (59.52%)
Offer ICU beds for admission	103 (61.31%)
Start home outreach services	61 (36.31%)
I don't think they can contribute in this domain	6 (3.57%)
Other (please specify)	12 (7.14%)
**Q5. How do you think the private sector could contribute toward consumables/supplies? [Multiple responses**. ***N*** **=** **168]**	
Masks	101 (65.48%)
Drugs	89 (52.98%)
Testing kits	103 (61.31%)
Gloves and protective gear	115 (68.45%)
Not sure	9 (5.36%)
I don't think they can contribute in this domain	28 (16.67%)
Other (please specify)	7 (4.17%)
**Q6. How do you think the private sector could contribute financially?[Multiple responses**, ***N*** **=** **168]**	
Contribution directly to local govt hospitals	51 (30.36%)
Provide free or subsidized treatment to those who cannot afford it	113 (67.26%)
Contribution to the PM fund	43 (25.60%)
I don't think they can contribute in this domain	20 (11.90%)
Other (please specify)	9 (5.36%)
**Q7. How do you think the private sector could contribute toward education?[Multiple responses**, ***N*** **=** **168]**	
Educating non ICU health care workers toward ICU services	96 (57.14%)
Training of healthcare professionals for management of non-sick COVID-19 patients	121 (72.02%)
Training of other healthcare professionals toward personal protection and safety	118 (70.24%)
Training of students (MBBS) toward volunteer work for COVID-19	84 (50.00%)
Training non MBBS students toward volunteer work for COVID-19	65 (38.69%)
Spread awareness amongst the population	119 (70.83%)
I don't think they can contribute in this domain	7 (4.17%)
Other (please specify)	3 (1.79%)
**Q8. How do you think the private sector could contribute toward care for non COVID-19 patients?[Multiple responses**, ***N*** **=** **168]**	
Virtual consulting	130 (77.38%)
Arranging drug delivery	71 (42.26%)
Admission of sick patients	108 (64.29%)
Arrange infusions for those due for infusions (e.g., blood transfusion, vaccines, biologics, cancer chemotherapy)	110 (65.48%)
I don't think they can contribute in this domain	4 (2.38%)
Other (please specify)	5 (2.98%)
**Q9. What form of virtual consulting do you think is the most cost and effort efficient in the Indian scenario?[Multiple responses**, ***N*** **=** **168]**	
Telephone	98 (58.33%)
Video	102 (60.71%)
Email	37 (22.02%)
I don't think virtual consulting is a good idea	19 (11.31%)
I am not sure	8 (4.76%)
Other (please specify)	2 (1.19%)
**Q10. Blood banks are facing an acute shortage these days. Would you like to be a blood donor?[Multiple responses**, ***N*** **=** **168]**	
Yes	126 (75.00%)
No	42 (25.00%)
**Q11. Have you contributed toward the pandemic in any way yet?[Multiple responses**, ***N*** **=** **168]**	
Financially	81 (48.21%)
Feeding the poor	50 (29.76%)
Tele consulting	86 (51.19%)
Going to job regularly (essential services)	116 (69.05%)
Volunteer work	26 (15.48%)
Education	71 (42.26%)
Other (please specify)	6 (3.57%)
**Q12. How many patients have you provided tele-consulting yet? [*****N*** **=** **13]**	
1–10	8 (61.54%)
11–20	2 (15.38%)
21–50	2 (15.38%)
51–100	1 (7.69%)
>100	0 (0.00%)
**Q13. Do you think you have contributed enough? [*****N*** **=** **91]**	
Yes	36 (39.56%)
No	34 (37.36%)
I am not sure	21 (23.08%)
**Q14. What concerns do you have regarding the current strategy the Government has employed? [Multiple responses**, ***N*** **=** **91]**	
Government authorities have not involved the private sector adequately.	53 (58.24%)
Private sector is involved too much, which could lead to a decrease in the healthcare given to patients of other conditions.	6 (6.59%)
Government does not have enough funding.	27 (29.67%)
Private sector is not part of the decision making process.	41 (45.05%)
I think they are handling it perfectly	18 (19.78%)
Other (please specify)	11 (12.09%)
**Suggestions**	***N*** **=** **41**
**Encourage moral values amongst the HCWs**	
Honesty	1
Volunteer for COVID-19 patient care	1
**Encourage more communication between the government and private sector**	
More help from the government to medical staff	1
Proper coordination	1
**Educate public**	
Explain safety measures to own family and patients	3
Educate oneself about covid-19 in order to give advice to those who require it	4
Dispel myths	2
**Research**	
Study patients currently under our care	1
**Train HCWs**	
Non urgent patient care taught to HCWs so that experienced individuals can treat severe COVID patients	2
**Donations**	1
**Patient Care**	
Treat COVID patients	9
Practice Telemedicine	7
Continue practice	5
Provide outstation medical services	1
Provide treatment free of cost for the poor	1
Formulate practical protocols for patient care	1

The survey had three pages, with a logics function applied two times, to segregate those respondents who thought private sector could contribute to research, and those who had delivered teleconsultations. Since short survey time is important for higher response rates, we tried to limit questions of certain sections to the relevant respondents using the logic tool on SurveyMonkey to skip to a specific question when not relevant. The first section began with a question asking for areas in which the respondents felt contributions toward the pandemic can be made by the private sector. According to their answers, they were directed to the corresponding questions. For example, when respondents were asked a question on ‘areas in which the private sector could contribute toward this pandemic,' those who answered ‘research' were directed to research related questions”.

Moreover, to reduce survey time, only first few questions were mandatory. The open ended question was non-mandatory. The average time to complete the survey was 5 min.

Content validity of the survey questionnaire was performed using Lawshe's method and confirmed by three professors and one undergraduate medical student. The validated survey questionnaire was pre-tested among five HCWs, and the identified errors in wording, grammar or syntax were rectified.

### Survey Dissemination

The survey was circulated on social media platforms (Twitter, Facebook, WhatsApp) with the hashtags #privatesector and #COVID. Besides, they were circulated widely among contacts of the author team on WhatsApp and other instant messaging platforms. Participation was voluntary and no incentives were offered for survey completion. The respondents could change the answers before submission but not after it.

### Data Anonymity and Analysis

Data collection was partly anonymised, with internet protocol addresses being the only linked identifier. These were handled by the first and corresponding author, while data shared with other authors and in the supplementary material of the manuscript was completely anonymised.

Internet Protocol addresses were checked to avoid duplication of responses. Descriptive statistics were performed, and the results were expressed as numbers (percentages). The data was downloaded from surveymonkey.com and figures drafted using Microsoft excel.

### Ethics Approval

Exemption from review was obtained from the institute ethics committee [2018-62-IP-EXP] as per local guidelines. We adhered to the Checklist for Reporting Results of Internet E-surveys to report the data (20).

## Results

The participants included doctors (84.62% of the 213 respondents) (age 35 years ± 11.1), nurses and paramedical staff (3.30%), and medical students, administrative, and laboratory staff (12.08%) practicing in private sector hospitals as defined in the methods section above. 42.86% were doctors from large corporate hospitals (>50 beds), 6.59% from smaller bedded hospitals (<50 beds), 17.58% ran individual practices (clinics or outpatient departments), and 41.76% were from teaching hospitals.

Nearly half (47.4%) felt that the contribution from the private medical sector has been suboptimal. Suggestions for improved contributions included patient care (71.8%) and provision of equipment (62.4%), and research (39.9%) (Figure 1).

Participants suggested increased involvement in screening (69.6%), testing (69.1%), intensive care (61.31%), and non-intensive-care (59.52%) beds. Some (36.3%) felt that effective home outreach services could also be provided.

Participants believed that the private healthcare sector could provide insights into new testing methods (67.2%), vaccines (60.3%), and new or repurposed drugs (55.2%). Most participants (67.3%) preferred use of their financial contribution for subsidized treatment of patients while only 31% favored donation to public agencies.

Most respondents felt that they could play a significant role in educating healthcare workers, medical students, and the community. Another area of deemed support was maintaining continuity of care for non-COVID-19 patients, using virtual consultation services (77.4%), teleconsultation being the preferred option (60%). Teleconsultations were being offered at the time of participation in the study by 13 doctors.

More than half (58.2%) felt a need for greater involvement of the private sector in the pandemic response including policy making. Nearly half of the participants had made monetary donations for the pandemic from their personal funds (48.2%). Over one-thirds (37.36%) felt that they wanted to contribute more toward the pandemic response, and as many as 75% were keen to donate blood.

## Discussion

In our e-survey assessing the opinions and readiness of HCWs in the private healthcare sector, we found that participants felt that they had not contributed enough and were positively inclined to participate in the pandemic response. They expressed readiness to participate in screening, testing, patient care, support for equipment and clinical trials of newer drugs as well as repurposed medicines, vaccines, or newer diagnostic tests.

While testing and tracing contacts remains the primary public health response to an infectious disease pandemic, over 3 million samples have been tested in India since January 2020 (21). Although we have attained testing capacity of 1 lac samples per day, it would still take more than three and a half years to test 10% of the population. This appears to be an optimistically conservative but inadequate strategy in a country with more than 1.3 billion susceptible individuals (22, 23). Collaborations between government and private healthcare centers can decentralize screening and testing facilities, offloading central agencies while increasing the capacity and outreach. The recent success of the 4 P model in Iran (public-private-people partnership) suggests that a similar approach, of classifying partnership into public-private, public-people, and private-people partnerships without restrictions may be fruitful toward seamless delivery of healthcare in these challenging times (24).

While the public sector has been holding forte in the past few months, the need for additional resources is being increasingly felt. The private healthcare sector has significant potential (25–27), with 58% of the hospitals, 29% of the beds, and 81% of doctors (28). Under severe strain, similar collaborations have been forged in Italy, Spain, and several other countries (29, 30). A similar exercise in India would be a prudent way ahead in these times.

A large number of blood banks are in the private healthcare sector in India, and it might be worthwhile to explore the conversion of private blood banks into specialized units for the promising convalescent plasma donation therapy, if efficacy is proven in ongoing clinical trials. This will not only tide over the ongoing acute shortage of blood products but also be a sustainable source of convalescent plasma for therapy in severe COVID-19 (31). In fact, a vast majority of respondents expressed their willingness to donate blood to tide over the acute shortage of blood products in present times. Moreover, the most significant hurdle in vaccine development is the time frame available (32). With a strong infrastructure support and the ability to obviate layers of bureaucracy, participation by the private sector may expedite these efforts. Moreover, in view of the large proportion of the population resorting to private healthcare in India, PPP may support existing trials to generate evidence base for safety and efficacy of the various drugs being explored for COVID-19 (33).

While most public facilities are busy in COVID-19 care, patient with *non-COVID* ailments have faced neglect and apathy. Private healthcare respondents are willing and prepared to participate. Additionally, a forward triage protocol using tele-medicine services may in-fact herald a revolution for a large number of technology-enabled non-COVID patients (34). In the Western world too, teleconsultations are being increasingly preferred as means of avoiding congestion in public spaces (35). The recent descriptions of an adaptable crisis management digital platform in Canada that dramatically streamlined patient intake, triage, monitoring, referral, and delivery of non-hospital services suggest potential utility in this model (18). By obviating layers of bureaucracy prevalent in the public sector in India, a strong backbone of available infrastructure in the corporate private healthcare systems may offer the distinct advantage of rapid and widespread implementation of these services. Community outreach programmes may further enhance percolation to the rural community. Such programmes can offer routine healthcare, as well as cater to prevalent anxiety and mental health illnesses, while enhancing preparedness for a disaster like situation in a country that is lagging behind many others on the epidemic curve (36). The willingness of private healthcare workers to participate is encouraging toward planning and exploring these options further. Although lower literacy levels and traditional patterns of doctor-patient interactions are a challenge in providing effective home-based outreach care in India, yet the scope of mobile networks and empowerment by these hand-held computers cannot be underestimated (37, 38).

Nearly two-thirds of the respondents felt that the private sector could leverage its financial resources by providing free or subsidized treatment to patients. While the Government makes efforts to meet the requirement of ventilators, stuttering from the onslaught of paused supply from Europe and China, it is prudent to recognize and utilize the dormant resources lying in the private hospital intensive care (39–41). Further, private laboratories and research facilities, encouraged to develop new cost-effective and rapid high through-put testing methods, should start showing results soon.

In unusual times such as this, lessons could be learnt from past experiences. During the influenza pandemic of 2009, all dealings in India were restricted to the public sector to keep track of cases while ensuring affordable healthcare. This eventually led to an infrastructural deficit, and the consequent need to amend policies to include support from the private sector (42). Such experiences from previous epidemics have probably contributed to our HCWs believing that the private sector in India can participate in a more significant manner.

### Strengths and Limitations

This is the first survey including a wide set of stakeholders in the private healthcare sector and in our opinion, it is an important move in the right direction to ascertain willingness and preparedness.

Although only a fraction of those approached consented to be a part of the survey, the results, subject to opinionated biases of a small set of young technology-empowered respondents, largely doctors, are enlightening and encouraging. Further, since the study design was that of an exploratory study using electronic methods to distribute the survey, it lacks the rigor of a stratified sampling technique with a statistically calculated sample size. However, we highlight the need to conduct larger population based studies on the subject. In addition, despite it having the advantage of a diverse representation of voices across the country, opinions may be influenced by a differential approach determined by local state policy.

Since this study design was of convenience using social media tools, in order to be helpful in public policy matters, especially given the diversity of the Indian healthcare services system throughout the country, data in future efforts may be captured under careful definition, appropriately stratified to region, city-tier, rural and urban characteristics and the like. This would offer further insights into the true and strong presence of the private sector where their participation would be needed the most. However, the encouraging results from this preliminary report may be used to design further studies to form the basis for public policy action.

## Conclusion

In the face of an unprecedented disease, with mystical transmissibility and unprecedented ability to devastate the human population, it is not surprising that the public healthcare sector is under more stress than it can handle alone. We have a large private healthcare sector in our country which is not only equipped but also willing, to share the burden of disease. We have identified an encouraging response from the private health sector in contributions toward the pandemic. Furthermore, there is a positive attitude toward teleconsultation services, which bridge the gap for non-COVID care in the pandemic period. Thus, a pragmatic approach to facilitate the private-public partnership may go a long way in mitigating the community impact and reduce mortality in current times. An open, healthy and swift discussion between the public and private sector should be the first step toward sorting gray areas.

## Data Availability Statement

The datasets presented in this study can be found in online repositories. The names of the repository/repositories and accession number(s) can be found in the article/supplementary material.

## Ethics Statement

The studies involving human participants were reviewed and approved by Sanjay Gandhi Postgraduate Institute of Medical Sciences [2018-62-IP-EXP]. Written informed consent for participation was not required for this study in accordance with the national legislation and the institutional requirements.

## Disclosure

This paper was submitted to MedRxiv, the pre-print server for health sciences.

## Author Contributions

All authors listed have made a substantial, direct and intellectual contribution to the work, and approved it for publication.

## Conflict of Interest

The authors declare that the research was conducted in the absence of any commercial or financial relationships that could be construed as a potential conflict of interest.
